# Significant Land Cover Transitions and Regional Acceleration at the Continental Scale of Africa over the Last Four Decades

**DOI:** 10.3390/s26082318

**Published:** 2026-04-09

**Authors:** Hidayat Ullah, Wilson Kalisa, Shawkat Ali, Delong Kong, Jiahua Zhang

**Affiliations:** 1 Space Information and Big Earth Data Research Center, School of Computer Science and Technology, Qingdao University, Qingdao 266071, China; ullahhidayat@qdu.edu.cn (H.U.); shawkat@qdu.edu.cn (S.A.); kongdelong@qdu.edu.cn (D.K.); 2 Key Laboratory of Digital Earth Science, Aerospace Information Research Institute, Chinese Academy of Sciences, Beijing 100094, China

**Keywords:** land cover change, remote sensing, Intensity Analysis, spatiotemporal dynamics, forest degradation, Africa

## Abstract

Land cover (LC) change is reshaping terrestrial ecosystems and profoundly impacting sustainable development in Africa, yet the long-term, continental-scale spatiotemporal dynamics of these shifts remain obscured. To address the above issue, this study systematically explores the spatiotemporal dynamics of LC across Africa from 1985 to 2022 by leveraging the fine-resolution remote-sensing-derived GLC_FCS30D LC dataset within a stratified Intensity Analysis framework. To decompose landscape changes into interval, category, and transition levels across five climatic sub-regions of Africa, we systematically evaluate the temporal consistency of land systems. This hierarchical approach disentangles systematic transition pathways from random fluctuations, thereby revealing the distinct regional regimes governing continental transformation of LC. Our results ultimately show a strong LC change acceleration in Africa after 2010, mainly in Southern, Eastern, and Western Africa, which together made up 80 to 90% of the continent’s LC dynamics. During the whole study period, shrubland and grassland had the highest gross turnover due to their high bidirectional volatility. Intensity-wise, forest remained inactive even though it was a persistent net loser to crop in East Africa (2010–2020), to shrub in Southern Africa (1990–2022), and to wetland in West Africa during the post-2000 intervals. Wetland had a major change in dynamics from historical growth during 1985–1990 to systematic decline in 2015–2022. Cropland increased by systematically targeting shrubland and grassland, mainly in East Africa. Additionally, the Sahel contributed 40% of continental grassland to bare area transitions, despite some recovery of grassland in the region. These findings show that aggregate net-change metrics obscure the volatility in African LC; therefore, distinct regional regimes such as agricultural expansion and forest degradation necessitate spatially differentiated management strategies.

## 1. Introduction

Africa represents a critical frontier for global land system science, where a rapidly growing population and extensive terrestrial ecosystems interact under intensifying climatic and anthropogenic pressures. These shifts are profoundly reshaping terrestrial carbon stocks, disrupting hydrological cycles, and challenging food security, thereby threatening the continent’s trajectory toward the UN Sustainable Development Goals. However, the ability to resolve the continental-scale mechanisms driving these land cover (LC) dynamics remains constrained by persistent spatial, temporal, and methodological limitations. To address this, a growing body of literature has attempted to document the nature, extent, and drivers of these transformations across varying scales [1,2,3,4,5]. At the continental level, Midekisa et al. leveraged Google Earth Engine (GEE) to map land cover (LC) and impervious surface changes from 2000 to 2015 [1]. More recently, Masolele et al. utilized high-resolution Planet NICFI data to produce a comprehensive assessment of post-deforestation land use from 2001 to 2020 [2]. Complementing these continental efforts, regional assessments have provided further granularity. In West Africa, Herrmann et al. conducted multi-decadal analyses of regional spatiotemporal change [3]. Similarly, in East Africa, Brink et al. analyzed dynamics across the IGAD region using medium-resolution imagery, and Bullock et al. quantified three decades of transition hotspots and totals [4,5]. Despite these advances, a unified analysis that systematically integrates high-resolution spatial detail with long-term temporal consistency across the entire continent remains a significant gap.

The rapid maturation of global remote sensing products has shifted the scale of what is possible in monitoring terrestrial ecosystems. While earlier studies used coarse resolution (AVHRR/MODIS), the opening of the Landsat archive and GEE now allow high-resolution analysis and enable their application at continental and global scales for the first time, also at very high resolutions [6,7,8,9]. Consequently, we now have access to consistent, long-term global products, such as the ESA Climate Change Initiative LC, GlobeLand30, and, crucially for this study, GLC_FCS30D, a global 30 m land cover dynamics product derived from dense Landsat time-series (TM, ETM+, OLI) using a continuous change-detection method, providing 35 fine-grained LC classes from 1985 to 2022 [10,11,12].

The Intensity Analysis framework proposed by Aldwaik and Pontius [13] provides a hierarchical approach that quantifies change at different levels and has been used in many spatiotemporal LC change studies [14,15]. This method provides a more rigorous depth of analysis. Deciphering the complexities of African LC requires such an inclusive framework, one capable of systematically unifying the spatial and temporal dimensions of LC change. While Intensity Analysis has been employed in recent African studies, these efforts and previous attempted methods for LC change analysis have been predominantly circumscribed by sub-regional boundaries, single-category focuses, or the limitations of coarse-resolution datasets [16,17,18,19,20,21]. Consequently, a unified implementation of this framework that integrates high-resolution spatial detail with long-term temporal consistency is required to fully capture Africa’s comprehensive land system trajectories.

Building on progress in remote sensing and methods, and to address remaining challenges related to spatial and temporal coverage as well as application of methods to LC change, we present here the first continental, long-term (1985–2022) high-resolution Intensity Analysis of LC change for Africa, and the disaggregation by the Intergovernmental Panel on Climate Change Fifth Assessment Report (IPCC AR5) [22]. The specific objectives of this study are as follows: (1) to quantify interval-level change intensities and their temporal dynamics across Africa from 1985 to 2022; (2) to characterize category-level gains and losses and to identify which LC classes are systematically active versus dormant; (3) to elucidate transition-level targeting and avoidance patterns, revealing which conversion pathways are systematic versus random; and (4) to integrate these metrics within a stratified framework to map distinct “dominant landscape trajectories” (e.g., deforestation, forest degradation, desertification). By systematically decomposing change intensities across the five IPCC AR5 sub-regions, this study identifies specific geographic hotspots where systematic LC conversions are most extensive in both intensity and area. This continental and sub-region stratified approach was designed to inform specific areas where targeted actions are needed to halt and reverse ongoing LC change and to address a long-standing knowledge gap by providing a unified, methodologically rigorous basis for comparison of LC dynamics across Africa’s heterogeneous landscapes and their direct translation into continental development agendas and global sustainability goals.

## 2. Materials and Methods

### 2.1. Study Area

**Figure 1:**
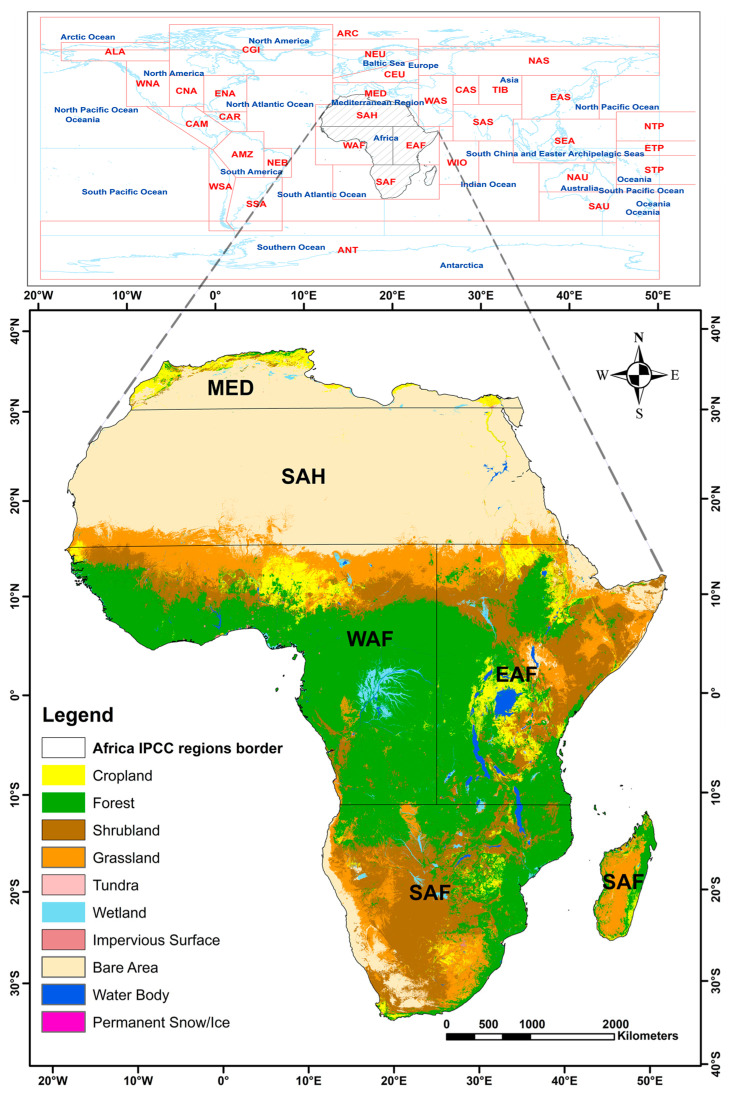
Map of the study area (Africa) showing its global location and the boundaries of the five IPCC AR5 climate reference regions. The land cover distribution shown is the observed classification for the year 2022, based on the reclassified GLC_FCS30D product (Table 1).

Africa is the world’s second-largest continent, covering an area of about 30.3 million km^2^. It is also a global reservoir of biodiversity, hosting eight of the world’s 36 recognized biodiversity hotspots [23,24]. However, these ecosystems are increasingly threatened by human-induced pressures such as land-use change, large-scale deforestation and increasing urbanization [2,3,4]. The continent is home to a population exceeding 1.4 billion, and the majority of this population still depends on natural resources for subsistence, which has led to a resource scramble between cropland expansion, biomass energy use, and ecosystem conservation [25,26]. The combination of climatic influences and human land-use pressures is strongly spatially heterogeneous across the continent [27,28]. Therefore, this study adopts a stratified approach by dividing the continent into five regions based on the IPCC AR5 climate reference framework [22]. This zonation (Figure 1) allows for a more nuanced understanding of the LC dynamics, integrated with existing climate science frameworks.

The Mediterranean (MED) region is considered a “hotspot” of climate change, with rates of warming already exceeding global averages that aggravate drought stress on remaining rainfed agricultural systems and on remaining Mediterranean forests [29,30]. The hyper-arid Sahara Desert and semi-arid Sahara-Sahel (SAH) zones provide a unique climatic interface for land system dynamics where vegetation cover is largely modulated by the West African Monsoon [31]. West Africa (WAF) exhibits a strong latitudinal rainfall gradient, transitioning from Sahelian savannas to humid Guinean forests [32]. Defined by the high-altitude and topographic complexity of the Rift Valley system, East Africa (EAF) vegetation patterns are underpinned by a diverse array of microclimates [5]. Finally, Southern Africa (SAF), which is home to the largest dry tropical forest system in the world, has its vegetation productivity largely modulated by the El Niño-Southern Oscillation [33].

**Table 1:** Land cover Level-1 reclassification scheme [12].

ID	Reclassified Class	Abbrev.	Original Classes	Codes
**1**	Cropland	CRP	Rainfed, Irrigated, Paddy, Greenhouse	10, 11, 12, 20
**2**	Forest	FST	Evergreen/Deciduous broadleaf/needleleaf, Mixed	51–92
**3**	Shrubland	SHR	Shrubland, Evergreen/Deciduous shrubland	120, 121, 122
**4**	Grassland	GRS	Grassland	130
**5**	Tundra	TUD	Lichens and mosses	140
**6**	Wetland	WET	Swamp, Marsh, Peatland, Flooded flat, etc.	181–187
**7**	Impervious Surface	IMP	Impervious surface	190
**8**	Bare Area	BAL	Sparse vegetation, Bare rock, Soil, Sand	150, 152, 153, 200–202
**9**	Water Body	WTR	Permanent water body	210
**10**	Permanent Snow/Ice	PSI	Permanent snow and ice	220

### 2.2. Dataset

The primary LC dataset utilized for this spatiotemporal analysis is the Global LC with Fine Classification System at 30 m (GLC_FCS30D) [12]. This LC product addresses multi-sensor harmonization internally: it integrates data from Landsat 5, 7, 8, and 9 using consistent surface reflectance processing and a continuous change-detection algorithm that accounts for sensor-to-sensor spectral differences across the full 1985–2022 archive. Our study, therefore, relies on this pre-harmonized product rather than performing independent sensor-level processing. The dataset reports a high overall accuracy of 80.88% for the basic classification system, validated against 84,526 globally distributed reference samples using a stratified random sampling design [12]. At the level of the ten aggregated classes used in this study (Table 1), accuracy is expected to be higher than the 35-class fine classification, as aggregation reduces inter-class confusion (e.g., merging evergreen and deciduous forest sub-types into a single Forest class). This classification system is particularly crucial for capturing the heterogeneous transition zones characteristic of African landscapes. Such areas (fragmented mosaics of the Sahelian savannas and the rapid peri-urban growth in West Africa) are often oversimplified by coarser resolution LC datasets.

To construct a long-term trajectory of LC change spanning 37 years, we integrated two distinct temporal modules of the GLC_FCS30D product. For the pre-2000 era, we utilized the 5-year data maps (1985, 1990, and 1995), which are the only maps in the dataset temporally available during the pre-2000 era. For the post-2000 period, despite the availability of annual classification maps from 2000 to 2022, we used primarily 5-year steps to minimize interval bias but retained the final 2020–2022 interval to capture the most recent dynamics. This final interval spans only two years because 2022 is the last available year in the GLC_FCS30D product. While this shorter duration could amplify annualized intensity values relative to 5-year intervals, the Intensity Analysis framework explicitly accounts for interval duration in all intensity calculations (Equations (1)–(9)), normalizing change rates to a per-year basis and thus preserving cross-interval comparability [13,14]. This approach is scientifically justified by the need to mitigate “time interval bias,” a phenomenon where varying observation durations can artificially inflate or deflate annualized rates of change, rendering cross-temporal comparisons invalid [13]. Furthermore, while the GLC_FCS30D product incorporates temporal smoothing, annual LC maps inevitably contain residual inter-annual classification noise called “flicker,” which is mainly driven by phenological variability or atmospheric noise rather than true LC conversion [12]. By adopting a 5-year interval, we effectively apply a temporal filter, which allows focusing on stable, long-term land system changes and disregards short-term, intermediate changes, which may not be due to real transitions but to transitional uncertainty in the classifications.

Eventually, the dataset was harmonized to establish nine discrete time points (T1 to T9): 1985, 1990, 1995, 2000, 2005, 2010, 2015, 2020, and 2022. This selection enables a comparative analysis of 15-year trends in the late 20th century against the accelerated dynamics observed in the first 22 years of the 21st century. To mitigate the complexity of the original 35 fine-grained classes, we aggregated them into ten major LC categories following the Level-1 hierarchical grouping defined in the original dataset documentation [12]. This aggregation groups sub-classes that share the same dominant land surface characteristics and spectral signatures at the Landsat scale: for example, evergreen broadleaf, deciduous broadleaf, evergreen needleleaf, deciduous needleleaf, and mixed forest (codes 51–92) are merged into a single Forest class, as these sub-types are frequently confused in pixel-level classification due to overlapping spectral–temporal profiles, particularly in the transitional zones of African woodlands [12]. Similarly, the seven wetland sub-types (swamp, marsh, flooded flat, etc.) share inundation-related spectral characteristics that justify their aggregation. This Level-1 scheme is consistent with the IPCC land-use reporting categories and has been adopted in prior continental-scale LC assessments [7,11]. Importantly, the Intensity Analysis framework requires discrete, mutually exclusive categories with sufficient spatial representation to produce statistically meaningful transition matrices; retaining 35 classes at the continental scale would result in sparse matrices dominated by zero-valued cells, undermining the statistical power of the targeting tests. As a consequence, transitions occurring between fine sub-classes within the same aggregated category (e.g., evergreen forest converting to deciduous forest) are absorbed into the persistence diagonal and are not captured as transitions in the Intensity Analysis.

### 2.3. Method

To quantify the magnitude and rate of LC change, this study employs a spatiotemporally stratified adaptation of the Intensity Analysis framework [13]. While standard Intensity Analysis unifies the measurement of category size and stationarity across three hierarchical levels (interval, category, and transition), we extended this framework to explicitly account for Africa’s heterogeneous climate regimes. Specifically, we incorporated a spatial stratification layer, enabling continental land cover dynamics to be decomposed and consistently attributed to the five IPCC AR5 African sub-regions at each level of the Intensity Analysis.

Prior to analysis, cross-tabulation matrices were generated using Google Earth Engine (GEE) for each region and time interval. The GLC_FCS30D data were loaded from the GEE data catalog (projects/sat-io/open-datasets/GLC-FCS30D), reclassified according to the original paper scheme in Table 1, and clipped to each of the five IPCC AR5-defined African regions. Transition matrices were constructed for eight time intervals defined by the years $Y_{t}$: 1985–1990, 1990–1995, 1995–2000, 2000–2005, 2005–2010, 2010–2015, 2015–2020, and 2020–2022. Let $C_{tij}$ denote the area of transition from category i to category j during the interval $\left[Y_{t},Y_{t+1}\right]$, and let $\Delta Y_{t}=Y_{t+1}-Y_{t}$ be the duration of the interval in years.

The interval-level analysis examines the overall speed of change across different time periods. We calculated the annual change intensity ($S_{t}$) for each interval t and compared it to a uniform annual change intensity (U), which represents the hypothetical rate if change were distributed evenly across the entire study duration (1985–2022).

(1) $$S_{t}=\frac{\sum_{j=1}^{J}\sum_{i\neq j}C_{tij}}{\Delta Y_{t}\sum_{j=1}^{J}\sum_{i=1}^{J}C_{tij}}\times 100\%$$

(2) $$U=\frac{\sum_{t=1}^{T-1}\sum_{j=1}^{J}\sum_{i\neq j}C_{tij}}{\sum_{t=1}^{T-1}\left(Y_{t+1}-Y_{t}\right)\sum_{j=1}^{J}\sum_{i=1}^{J}C_{tij}}\times 100\%$$

where if $S_{t}>U$, changes in interval t are considered relatively fast; otherwise, they are slow. To operationalize the stratified framework, we decomposed the total change area for each interval into the contributions of the five sub-regions ($R_{k}$, where k∈{EAF,MED,SAF,SAH,WAF}). The regional contribution ($P_{t,k}$) was calculated as:

(3) $$P_{t,k}=\frac{C_{t,k}}{C_{t,total}}\times 100\%$$

where $C_{t,k}$ is the gross change area in region k and $C_{t,total}$ is the aggregate change area for Africa during interval t.

The category-level analysis evaluates whether specific LC classes were active or dormant during a given interval. We calculated the annual gross gain intensity ($G_{tj}$) and annual gross loss intensity ($L_{ti}$) for each category.

(4) $$G_{tj}=\frac{\sum_{i\neq j}C_{tij}}{\Delta Y_{t}\cdot C_{t\cdot j}}\times 100\%$$

(5) $$L_{ti}=\frac{\sum_{j\neq i}C_{tij}}{\Delta Y_{t}\cdot C_{ti\cdot }}\times 100\%$$

Here, $C_{t\cdot j}$ is the size of category j at the end of the interval ($Y_{t+1}$), and $C_{ti\cdot }$ is the size of category i at the start of the interval ($Y_{t}$). These intensities were compared to the overall annual change intensity for that interval ($S_{t}$, Equation (1)). A category is considered an active gainer if $G_{tj}>S_{t}$ and an active loser if $L_{ti}>S_{t}$. To spatially attribute these category-level volatilities, we applied stratified decomposition to the total gross gain area ($A_{\mathrm{Gain},t,j}$) and gross loss area ($A_{\mathrm{Loss},t,i}$) of each category into the proportional contributions of the five sub-regions (k). The stratified contribution of each region to gross gain ($P_{\mathrm{Gain},t,k,j}$) and gross loss ($P_{\mathrm{Loss},t,k,i}$) was calculated as:

(6) $$P_{\mathrm{Gain},t,k,j}=\frac{A_{\mathrm{Gain},t,j,k}}{A_{\mathrm{Gain},t,j}}\times 100\%$$

(7) $$P_{\mathrm{Loss},t,k,i}=\frac{A_{\mathrm{Loss},t,i,k}}{A_{\mathrm{Loss},t,i}}\times 100\%$$

where $A_{\mathrm{Gain},t,j,k}$ is the gross gain area of category j within region k during the interval time t, and $A_{\mathrm{Gain},t,j}$ is the gross gain of category j for the entire continent during that same interval.

The transition level analysis characterizes the transitions between categories based on the condition if changes are targeted or avoided. For each gaining category n, we examined the intensity of transition from category m ($R_{tmn}$) relative to a uniform transition intensity ($W_{tn}$).

(8) $$R_{tmn}=\frac{C_{tmn}}{\Delta Y_{t}\cdot C_{tm\cdot }}\times 100\%$$

(9) $$W_{tn}=\frac{\sum_{i\neq n}C_{tin}}{\Delta Y_{t}\sum_{i\neq n}C_{ti\cdot }}\times 100\%$$

where $W_{tn}$ represents the intensity if category n gained uniformly from all available non-n categories. If $R_{tmn}>W_{tn}$, the transition from m to n is targeted; if $R_{tmn}<W_{tn}$, it is avoided. Finally, to identify the specific geographic engines of these systematic pathways, we applied the stratified analysis at the transition level. We decomposed the area of each transition from category m to n ($C_{t,mn}$) into its regional components. The proportional contribution of region k to a specific transition ($P_{\mathrm{trans},t,k,mn}$) was derived as:

(10) $$P_{\mathrm{trans},t,k,mn}=\frac{C_{t,mn,k}}{C_{t,mn}}\times 100\%$$

where $C_{t,mn,k}$ represents the area of transition from m to n occurring within region k during interval t, and $C_{t,mn}$ is the total area of that transition across Africa.

To complement the statistical Intensity Analysis with an explicit spatial representation, the most significant major LC transitions were mapped across the African continent for the full study period. These major transitions were selected based on transition-level Intensity Analysis results, specifically identifying category pairs that exhibited (i) a high frequency of targeted status $R_{tmn}>W_{tn}$ and (ii) contributed substantial cumulative area by 2022. We then mapped the 1985–2022 conversions associated with these transition pathways.

The analytical workflow proceeded in three stages: (1) cross-tabulation matrices were generated in Google Earth Engine by computing pixel-level transitions between consecutive time points for each of the five sub-regions at 30 m resolution, yielding 40 region–interval matrices (5 regions × 8 intervals); (2) these matrices were exported as CSV files and ingested into a Python 3.10-based implementation of the Intensity Analysis framework; (3) interval-, category-, and transition-level metrics were computed and the regional decomposition was applied at each level. The complete computational pipeline (including the GEE processing scripts, the Python-based Intensity Analysis implementation, and all intermediate transition matrices) is publicly available at: https://github.com/hidayathaider35/Africa-LC-Intensity-Analysis.git (accessed on 24 March 2026).

## 3. Results

The following sections present the continental-scale results with integrated regional decomposition. To facilitate detailed examination of individual regional dynamics, per-region interval, category, and transition-level results are provided in the Supplementary Materials (Figures S1–S8).

### 3.1. Interval-Level Analysis of LC Change over Africa

**Figure 2:**
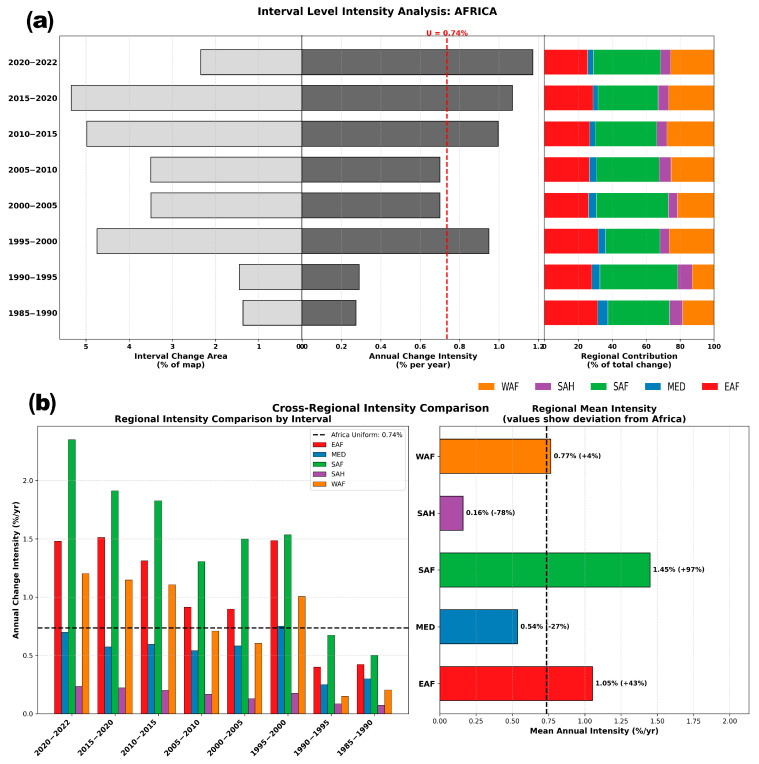
Interval-level stratified Intensity Analysis of LC change in Africa during 1985–2022. (**a**) The left panel shows the gross change area per interval as a percentage of the total map size. The center panel displays the annual change intensity for each interval compared to the U intensity. The right panel decomposes the total change area into the proportional contributions of five sub-regions. (**b**) Comparison of regional annual change intensities. The left subplot details intensity fluctuations by interval for each region, while the right subplot summarizes the mean annual intensity and its deviation from the continental baseline (WAF: West Africa, SAH: Sahara-Sahel, SAF: Southern Africa, MED: Mediterranean, EAF: East Africa).

For interval-level analysis results (Figure 2a), a uniform annual change intensity (U) of 0.74% was calculated over the full time period at the continental scale, which was used as a baseline for comparing temporal variation. Intensities were low in 1985–1995 (0.27–0.29%), rose sharply in 1995–2000 (0.95%), moderated in 2000–2010, and then accelerated after 2010, peaking in 2020–2022 (1.17%). EAF, SAF, and WAF accounted for ~80–90% of gross change in all intervals, with SAF contributing the largest share. In contrast, the SAH and MED regions contributed minimally to the gross change area. A cross-regional comparison of change intensities, shown in Figure 2b, highlights significant disparities in the rate of change among sub-regions, independent of their total area. SAF exhibited the most intense dynamics, with an average annual intensity of 1.45% (97% above U). EAF also experienced intense changes, with a mean intensity of 1.05% (+43% from U). WAF followed closely with the continental average (+4% deviation), and MED and SAH showed stagnant landscape dynamics, with intensities 27% and 78% below the continental baseline, respectively. Patterns of temporal synchrony of these intensities (Figure 2b) reveal that the acceleration observed in 2015–2022 was a co-occurring event across the three active regions (SAF, EAF and WAF), but the magnitude of this acceleration was disproportionately driven by SAF, where intensity was over 2.0% in the last interval (2020–2022).

### 3.2. Category-Level Analysis of LC Change in Africa

**Figure 3:**
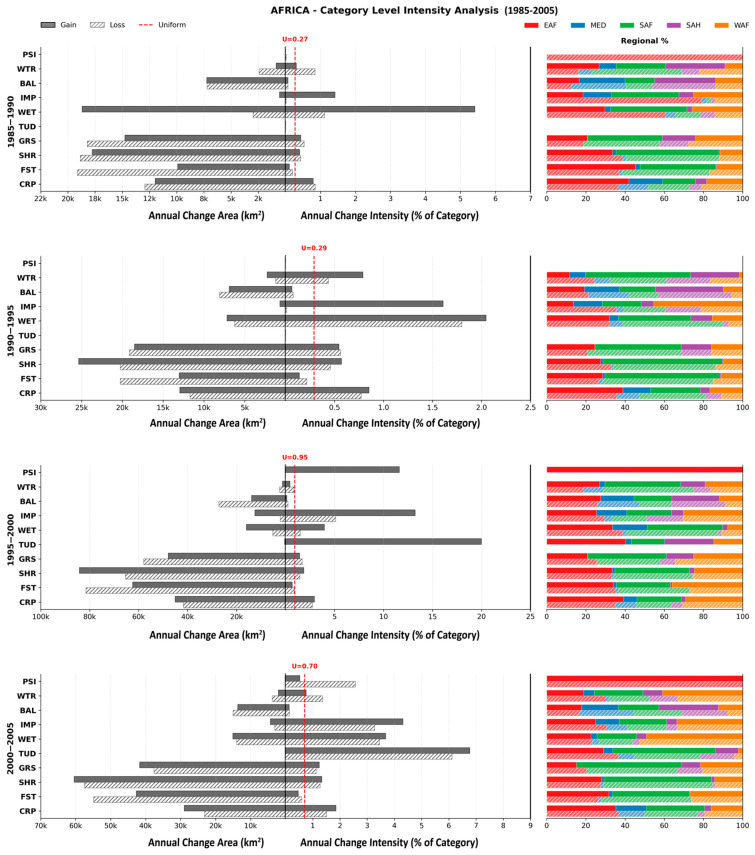
Category-level stratified Intensity Analysis for Africa from 1985 to 2005 (CRP: Cropland, FST: Forest, SHR: Shrubland, GRS: Grassland, TUD: Tundra, WET: Wetland, IMP: Impervious, BAL: Bare Area, WTR: Water Body, PSI: Snow/Ice).

**Figure 4:**
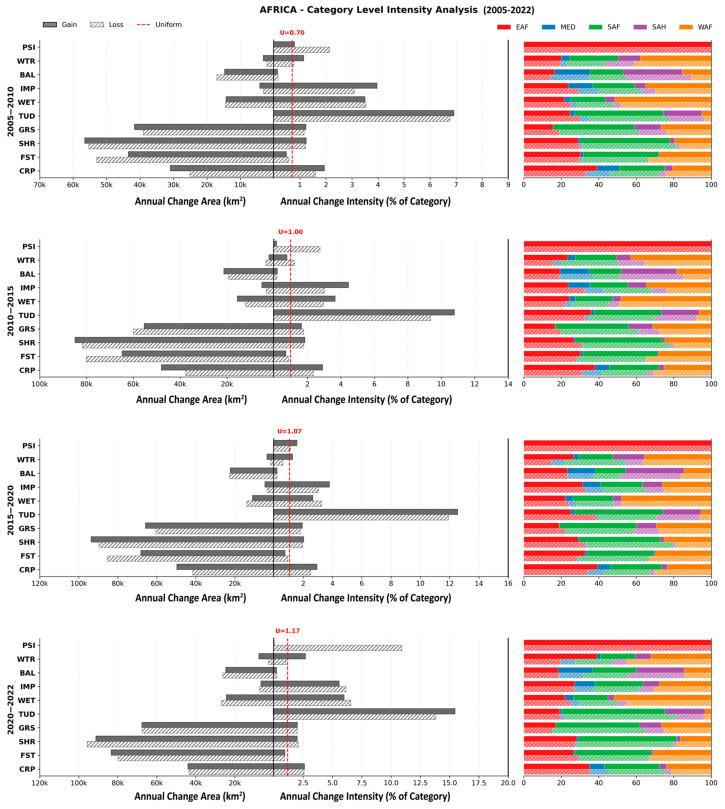
Category-level stratified Intensity Analysis for Africa from 2005 to 2022 (CRP: Cropland, FST: Forest, SHR: Shrubland, GRS: Grassland, TUD: Tundra, WET: Wetland, IMP: Impervious, BAL: Bare Area, WTR: Water Body, PSI: Snow/Ice).

The analysis results at the category level presented in Figure 3 and Figure 4 reveal a stark contrast between LC categories showing large area change and those showing high change intensity. In terms of annual change area, landscape dynamics were mostly dominated by four major vegetative categories, i.e., Shrubland (SHR), Forest (FST), Grassland (GRS), and Crop (CRP). SHR and GRS consistently showed the largest gross turnover, representing simultaneous gains and losses. This turnover was particularly high during the 2015–2020 interval, when SHR gross change exceeded 900,000 km^2^. The only two periods during which SHR experienced a net loss (active loss values exceeding active gain) were the initial interval (1985–1990) and the most recent interval (2020–2022). This massive gross change was overwhelmingly concentrated in SAF, which alone contributed 40–50% of both SHR active gains and losses across most intervals; yet, consistent net loss was observed in EAF. GRS displayed dominant loss over gains in pre-2000 intervals but shifted toward net gains or balanced dynamics in subsequent intervals (2000–2022).

CRP and FST displayed almost opposite behavior during the entire period. CRP was a systematic net gainer, as the area gained was much larger than the area lost in nearly all intervals, even though it was active both in gain and loss intensities. These gains originated predominantly from EAF across most intervals, while some losses were also observed in WAF. FST, on the other hand, was dormant in both gains and losses but functioned predominantly as a net loser, as its loss intensity values can be observed surpassing gain intensity in each interval, except for the most recent one. For instance, during the 2015–2020 interval, FST lost approximately 85,400 km^2^ per year while gaining only 68,100 km^2^. This consistent loss of FST was driven primarily by the SAF region and, to some extent, WAF. In some intervals, SAF and WAF collectively accounted for nearly 75% of the total continental FST loss while contributing less than 70% to FST gains. Bare Area (BAL) was the most dormant category throughout the entire period and had relatively smaller changes in each period and region.

In contrast to the spatially larger area categories, the smaller categories, such as Impervious (IMP), Wetland (WET) and Tundra (TUD), showed aggressive intensity whenever their gain or loss intensities surpassed the uniform rate, indicating that these LC types experienced the most rapid modification relative to their size. TUD (when it first appeared in all regions) recorded the highest intensity value of 20.1 during 1995–2000 among all categories in all intervals. The intensity of Snow/Ice (PSI) was somewhat similar to that of TUD in the sense that its intensity became active (only in gains) during 1995–2000; however, its dynamics were taking place exclusively within the EAF region.

IMP was an active gainer in all intervals, acting as a dominant active gainer with nearly no loss. The behavior of active gain and dormant loss was observed during the initial decade (1985–1995), but in the subsequent intervals, active gain continued to be larger than active loss; this unidirectional pattern broke in the most recent interval (2020–2022), where the magnitude of active loss was larger than active gain. In terms of region-wise change intensity for IMP, the pattern revealed repeated aggressive loss in EAF, revealing recovery in other regions, especially WAF. Water Body (WTR) showed alternating gain/loss phases, with losses concentrated in SAF/SAH during 2010–2015 and gains concentrated in EAF during 2015–2022. An interesting pattern can be noted for WET, where it was active in both gains and losses. It showed an aggressive expansion in the initial periods, starting with an intensity value of 5.5 in gains against 1.2 in losses during 1985–1990 (gains were about four times the losses). However, this ratio shrunk during the subsequent periods, and eventually active loss started to show higher intensity than active gains during 2015–2022. In the overall period and in recent intervals, these gradual dominant loss values were primarily attributable to EAF.

### 3.3. Transition-Level Analysis of LC Change over Africa

#### 3.3.1. Transition-Level Stratified Intensity Analysis

**Figure 5:**
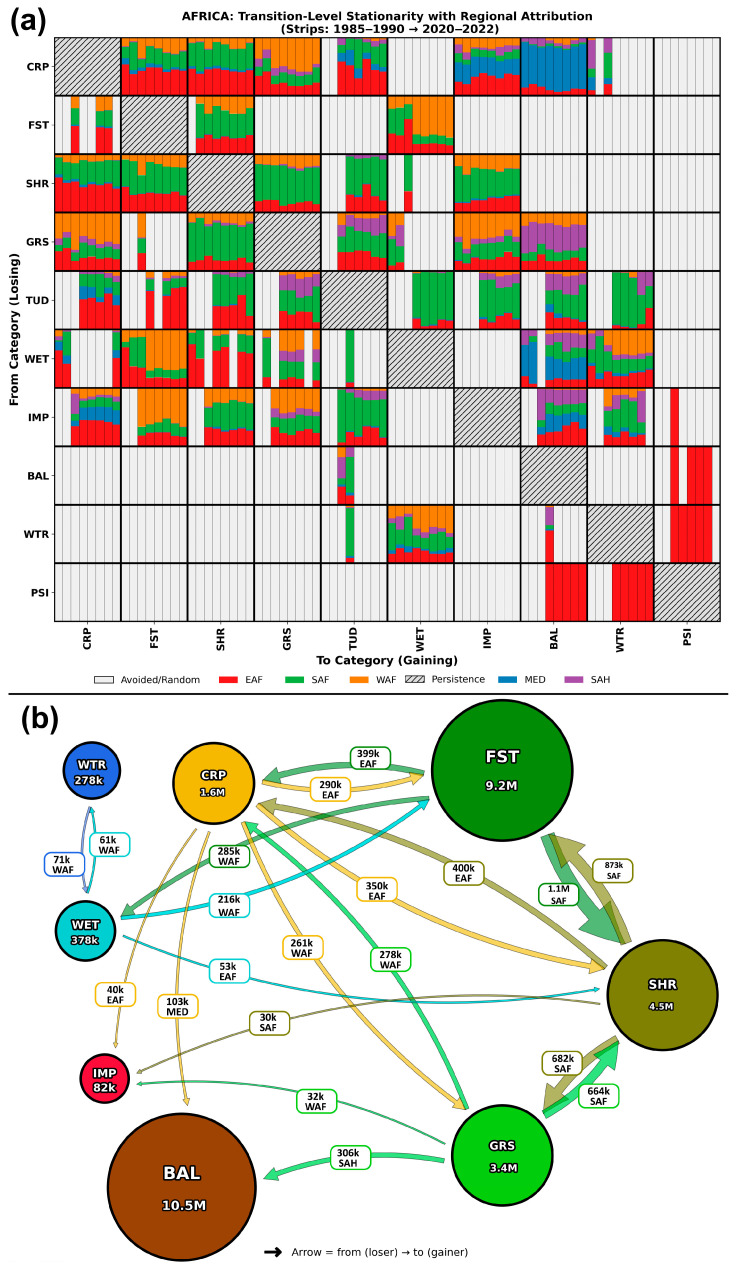
Transition-level analysis of targeted LC conversions in Africa (1985–2022). (**a**) Stationarity matrix showing targeted (colored) and avoided (gray) transitions across eight time intervals. Each off-diagonal cell contains eight vertical strips (1985–1990 through 2020–2022); colored strips indicate targeted transitions ($R_{tmn}>W_{tn}$), with colors denoting the dominant contributing sub-region. Diagonal cells (hatched) represent persistence. (**b**) Network representation of targeted transitions aggregated over the full study period. Nodes are proportional to mean class area; arrows point from the losing to the gaining category, with width proportional to cumulative transition area and color indicating the losing class. Labels show the transition area and the dominant contributing sub-region (CRP: Cropland, FST: Forest, SHR: Shrubland, GRS: Grassland, TUD: Tundra, WET: Wetland, IMP: Impervious, BAL: Bare Area, WTR: Water Body, PSI: Snow/Ice).

The transition-level stationarity matrix (Figure 5) provides a granular decomposition of the continental change budget, distinguishing between systematic LC conversions (targeted transitions) and those occurring disproportionately slowly relative to category abundance (avoided transitions). While the conversion of natural vegetation to CRP (SHR→CRP and GRS→CRP) was systematically targeted across all intervals, the stationarity matrix reveals that the reverse pathways (CRP→SHR and CRP→GRS) were equally persistent in their targeted status. Regionally, EAF and SAF dominated the SHR↔CRP exchange, whereas WAF dominated the GRS↔CRP exchange. The CRP transition relationships were nearly bidirectional with TUD, where dominant transitions were mostly observed in the post-2000 period (primarily in EAF followed by SAF). In contrast, WET and WTR exhibited unidirectional transition relationships with CRP: WET was targeted by CRP in the initial and recent intervals, while WTR targeted CRP during the initial intervals. The WET→CRP transition was observed predominantly in EAF and SAF, whereas CRP→WTR transitions occurred predominantly in SAH and SAF. Another strong unidirectional transition was observed when CRP was aggressively targeted by BAL (CRP →BAL) in the MED region (~80% of transition intensity) across all intervals; in return, CRP never achieved dominant recovery in any interval. IMP, on the other hand, targeted CRP throughout the overall period (1985–2022), yet recovery commenced only after the first decade of the study period (1995–2000); this transition intensity originated mostly from EAF (~50%), followed by equally distributed contributions from each remaining region.

Notably, the FST→CRP transition exhibited a distinct intermittent pattern, achieving targeted status only in specific intervals (e.g., 1995–2000 and 2010–2020). In return, FST was targeted by CRP (FST→CRP) in each interval transition; however, since the overall FST area is spatially enormous, FST being targeted implies that a substantial proportion of the FST area transitioned to CRP. Other categories exhibiting bidirectional transitions with FST include SHR and WET: the FST↔SHR transition (1990–2022) was observed approximately equally distributed across EAF, SAF, and WAF, while the FST↔WET transition was most evident in WAF, especially during the post-2000 period.

The matrix displays transitions from the “From” category (rows) to the “To” category (columns). Each cell contains eight bars representing the time intervals from 1985–1990 to 2020–2022. The height of the bar represents the proportion of the transition relative to the total change in the gaining category. The color fill indicates the contributing sub-region for transitions that were targeted (where transition intensity $R_{tmn}>$ uniform intensity $W_{tn}$). Gray bars indicate avoided ($R_{tmn}<W_{tn}$). To provide a synoptic overview of these transition-level results, Figure 5b presents a network diagram of the targeted transitions aggregated over the full study period (1985–2022). The dominant pathways include FST→SHR (1.1M km^2^, mainly in SAF), the bidirectional SHR↔GRS exchange (~665–683k km^2^, SAF), FST→CRP and SHR→CRP (~400k km^2^ each, EAF), and GRS→BAL (306k km^2^, SAH). The network clearly illustrates that SHR, GRS, FST, and CRP function as the central hub of African LC dynamics.

SHR and GRS exhibited mutually targeted transitions across all intervals. This transition was observed predominantly in SAF (~50% of transition intensity), followed by EAF and WAF (each contributing a mean of 20–25%). SHR also shared the same mutual targeted exchange with TUD during 2000–2022, primarily in EAF and SAF. This pattern of post-2000 bidirectional exchange was also evident between GRS and TUD in EAF, SAF, and some WAF. While GRS lost area to WET during the initial decade of the study period (1985–1995), it targeted WET in most intervals (five out of eight intervals). IMP and BAL consistently targeted GRS across all intervals, mainly in WAF, SAH, SAF and EAF regions. Even when GRS managed to recover from IMP by constantly targeting it from 1995 to 2000, GRS still failed to target BAL in any single period. TUD, having appeared in the 1995–2000 interval, consistently exhibited bidirectional transition exchanges with CRP (mainly in EAF), with IMP (in SAF), with SHR (dominantly in EAF and SAF), and with GRS (equally in EAF, SAF, and SAH). TUD was aggressively targeted by FST in EAF, by WET and WTR in SAF, and by BAL in almost every region.

A consistent transition between WTR↔WET occurred in WAF, followed by SAF and EAF, with smaller contributions from SAH and MED. WET was aggressively targeted by BAL across all regions, especially in MED; in return, WET failed to target BAL in any interval throughout the study period. In some intervals, WET was targeted by CRP, SHR, and GRS in almost all regions (except MED). IMP, as discussed with almost all of the above categories, is consistently targeted by these categories (except WET). Even though CRP in all regions, SHR and GRS in WAF, SAF and WAF were actively targeting IMP during the post-2000 period, IMP targeted these categories across all intervals (including pre-2000). However, IMP was aggressively targeted by FST in WAF, and by BAL and WTR in almost all regions during the post-2000 period. PSI exhibited mostly mutual/reciprocal relationships with BAL and WTR exclusively in EAF, with only a single interval (1995–2000) in which PSI targeted IMP.

#### 3.3.2. Dominant Spatial Transitions of LC in Africa

**Figure 6:**
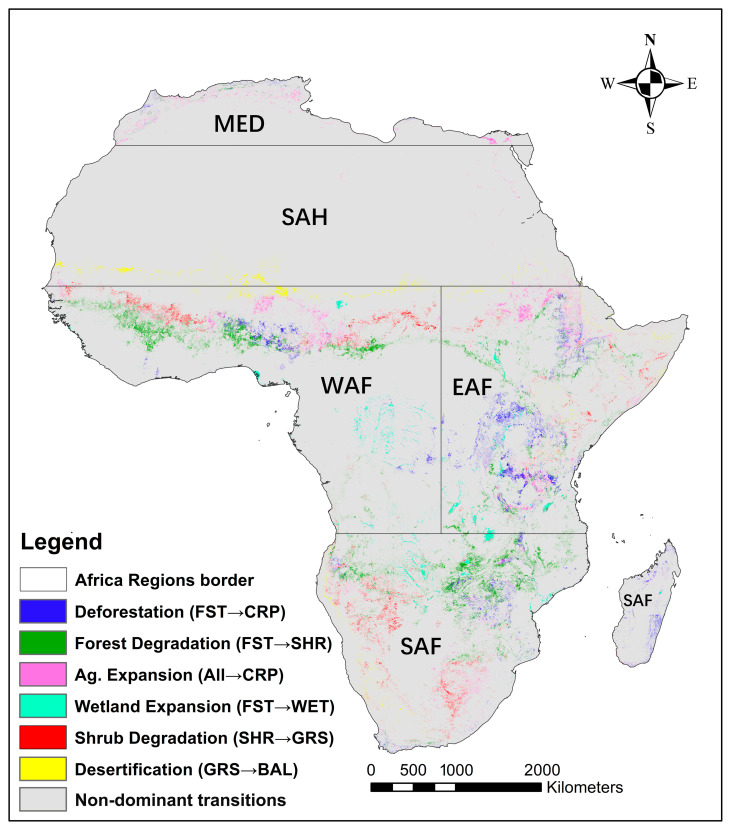
Map of the dominant LC transitions in Africa based on area.

To provide spatial context for the dominant LC transitions identified across Africa (Figure 6), we filtered the transitions based on two criteria: (i) consistent targeting behavior across multiple time intervals; and (ii) significant cumulative area representation in the final period (2022) of the study. Based on these transition-level targeting criteria, six dominant transition pathways were selected for cartographic representation: (1) Deforestation (FST→CRP), representing direct FST conversion to agriculture; (2) Forest Degradation (FST→SHR), indicating FST structural decline; (3) Agricultural Expansion (SHR/GRS/BAL→CRP), aggregating multiple pathways of CRP encroachment into natural vegetation; (4) WET Expansion (FST→WET), capturing FST-to-WET conversion; (5) Shrub Degradation (SHR→GRS), denoting vegetation structural simplification; and (6) Desertification (GRS→BAL), representing GRS conversion to BAL.

Deforestation was a widespread phenomenon across Africa, involving approximately 200,000 km^2^ of transition from FST to CRP. The EAF region was the main driver, accounting for more than half of this transition (106,000 km^2^), whereas SAF and WAF contributed approximately 20–25% each. Forest Degradation (FST→SHR) encompassed 459,000 km^2^ across the continent, of which 40% and 34% were attributed to SAF and WAF, respectively, while EAF accounted for 23%. Agricultural Expansion was dominant in EAF (95,000 km^2^ of the 227,000 km^2^ continental total), which represents 41% of total CRP expansion, followed by WAF, which contributed 25% in the SHR/GRS/BAL→CRP transition. WET Expansion occurred extensively (88,000 km^2^) and was mostly at the expense of FST cover (FST→WET); WAF contributed 36% to this process, while SAF and EAF contributed equally at 31% each. SHR→GRS Degradation entailed substantive vegetation replacement by GRS (166,000 km^2^) across Africa, which occurred predominantly in SAF (42%) and WAF (37%). Finally, Desertification was present continent-wide, with 90,000 km^2^ of area shifting from GRS→BAL. The SAH region accounted for 40% of this transition, while WAF and EAF accounted for 26% and 20%, respectively.

## 4. Discussion

### 4.1. Temporal Acceleration and Regional Drivers of LC Change over Africa

Interval-level intensities show low change in 1985–1995, an inflection in 1995–2000, and acceleration after 2010, consistent with the work of Potapov et al. [34], who observed that the global expansion of CRP accelerated since 2010, with Africa emerging as the dominant contributor. The synchronization of this acceleration across SAF, EAF, and WAF suggests that the operation of continent-wide drivers rather than isolated regional phenomena is consistent with the convergence of demographic momentum, economic liberalization, and increased integration into global commodity markets [35]. To contextualize this demographic dimension, Africa’s population approximately doubled from 550 million in 1985 to over 1.4 billion by 2022 [36], creating commensurate pressure on land resources for food production and settlement.

The marked 1995–2000 inflection point coincides with several transformative continental developments; most notably, the cessation of major civil conflicts in WAF and SAF, which catalyzed agricultural resettlement and the broader implementation of structural adjustment programs [37]. SAF’s disproportionate contribution to continental change intensity corroborates characterizations of the Miombo woodland belt as a principal arena of contemporary land transformation [33]. This dominance is consistent with the transition-level findings showing that SHR↔GRS and FST→SHR pathways are concentrated in this region and linked to the charcoal-driven energy economy supplying expanding urban centers [38].

The overall dormancy at the continental scale of SAH stands in stark contrast to narratives of desertification but is in agreement with the large body of evidence that the SAH has been experiencing a vegetation recovery in response to precipitation rebounds since the 1980s [39]. The transition-level analysis, however, indicates that this does not preclude targeted degradation at the local scale. In fact, the GRS→BAL transition was systematically targeted within SAH, indicating that even if vegetation change at the pixel level is limited, GRS-to-BAL soil transitions are still operational. This echoes Jiang et al., who emphasized the large spatial heterogeneity of Sahelian dynamics where broad-scale greening is intermixed with hotspot clusters of degradation stemming from multiple climatic and human drivers pulling in different directions [31].

### 4.2. Agricultural Expansion and Urban Encroachment Dynamics in Africa

Agricultural expansion remains the dominant transformation in our analysis. While agricultural production can increase through intensification measures such as sequential cropping and collaborative sustainable farming practices, the magnitude of the CRP net gain reported here suggests that extensive conversion is the main pathway of meeting growing food demand [40,41]. The EAF dominance in CRP gains corroborates Bullock et al., who reported an 18.15-million-hectare (34.8%) net CRP gain in seven East African countries (Uganda, Rwanda, Tanzania, Malawi, Zambia, Kenya, and Ethiopia) between 1988 and 2017, which took place mainly at the expense of open GRS, wooded GRS, and open FST [5]. Importantly, the transition-level analysis also showed that the reverse pathways (i.e., CRP→SHR and CRP→GRS) were consistently targeted, confirming that the agricultural expansion phase is marked by a significant degree of agricultural abandonment. This bidirectional exchange pattern agrees with land-use cycling in shifting cultivation systems reported by Tyukavina et al. in the Congo Basin and by Tong et al. in the West African Sudano-Sahelian zone [42,43]. The dynamics of simultaneous expansion and abandonment also suggest that agricultural net gains are the residue of much larger gross fluxes, with potential implications for carbon accounting in which agricultural expansion is treated as a unidirectional process [44]. However, while agriculture acts as the primary engine of vegetative conversion, it is simultaneously under pressure from the built environment. The consistent IMP expansion and its systematic targeting of CRP across all intervals and regions reflect Africa’s position as the most rapidly urbanizing continent. Africa experienced among the highest global urban expansion rates (3.9–4.7% annually in West Africa), exceeding population growth rates [45,46].

The aggressive, unidirectional, and exclusive CRP→BAL pathway in MED, without reciprocal recovery, reveals that agricultural lands in North Africa are degrading irreversibly. This is in line with Ali et al.’s projections of intensifying water stress in the Mediterranean rim, where warming rates are above global averages and precipitation declines threaten water-limited agricultural systems [30,47]. The asymmetry of this transition, with BAL consistently gaining from CRP and no dominant recovery being observed, suggests that the physical and climatic conditions of this region prevent the spontaneous recovery of agricultural lands that have become degraded, potentially leading to positive feedback between land degradation and agricultural productivity loss [48].

### 4.3. Forest Dynamics and Montane Ecosystems in Africa

Although FST appeared dormant at the category level, the transition-level decomposition of net changes in FST shows that its seeming dormancy is the result of offsetting gross fluxes (FST→CRP and FST→SHR repeatedly subtract area from FST; CRP→FST and SHR→FST only partly compensate). This finding aligns with Zhao et al., who confirmed that humid tropical forests in Africa are in a state of high flux, experiencing substantial loss and regrowth that effectively cancels out in net-change metrics, thereby masking the true extent of forest disturbance [49].

The regional attribution of FST losses reveals that SAF and WAF’s concentration of FST→SHR transitions reflects the charcoal production systems documented extensively in the Miombo literature. Muteya et al., for instance, reported deforestation rates of −1.51% per year in the Lubumbashi charcoal production basin (covering a little less than half of the SAF region), which were almost six times the national average of the Democratic Republic of the Congo, with a concomitant loss of Miombo woodland (from 78% to 40% cover) between 1990 and 2022 [38]. Meanwhile, in WAF, some studies recently uncovered that cocoa expansion is the main underlying driver of more than 37% of FST loss in protected areas in Côte d’Ivoire and 13% in Ghana, a planted area that is seriously underestimated by official reports [50]. Similarly, oil palm expansion is a major driver of deforestation in Central and West Africa, with production expanding 1.2 million hectares across sub-Saharan Africa since 1990 [51], and recent mapping revealing substantial forest loss attributable to oil palm in the Congo Basin countries [52]. In the GLC_FCS30D product, oil palm is not differentiated as a separate class and may be captured under Cropland (if recognized as a planted crop), Forest (if the canopy is sufficiently closed), or Shrubland (during early growth stages). Consequently, some portion of the FST→SHR and FST→CRP transitions in WAF may reflect oil palm establishment, while mature oil palm replacing natural forest would not register as a transition. This limitation underscores the need for crop-specific mapping layers to disentangle commodity-driven forest conversion in tropical West and Central Africa. In the broader Miombo belt, Sikuzani et al. found that the woody LC of Lualaba Province decreased from 62.9% in 1990 to below 25% in 2024, associated with an annual deforestation rate of −1.13% resulting from agricultural and mining fragmentation [53].

Unlike the continuous degradation observed in FST→SHR, the FST→CRP transition was episodic, concentrated in specific intervals (1995–2000 and 2010–2020). This underscores that reducing emissions from deforestation and forest degradation monitoring that overlooks degradation (e.g., by relying solely on deforestation detection) will miss significant loss in the Congo Basin [54,55]. In agreement, Tyukavina et al. similarly emphasized that about 84% of the Congo Basin forest disturbance can be attributed to small-scale clearing, which is not represented in other common mapped drivers such as selective logging (~10%) [43]. These forest loss dynamics have cascading effects on high-elevation sensitive ecosystems, particularly in EAF. The oscillating PSI trajectory with alternating loss and gain phases is likely reflecting multi-year precipitation variability superimposed on long-term warming trends. The absolute concentrations of TUD and PSI dynamics in EAF highlight the vulnerability of these high-elevation ecosystems to the combined pressures of warming and deforestation, which our results show is aggressively targeting the surrounding vegetation [56].

### 4.4. Shrubland-Grassland Dynamics and Savanna State Transitions Within Africa

The massive bidirectional flux of SHR and GRS with the mutually targeted SHR↔GRS exchange persisting across all intervals reflects the structural oscillation between woody and herbaceous vegetation states characteristic of African savannas. These dominant transitions in SAF may be associated with El Niño-Southern Oscillation fluctuations, with positive precipitation anomalies facilitating woody encroachment, while grass dominance is favored during dry periods due to mortality and fire [57,58]. These bidirectional dynamics could be reinforced by fire–vegetation–herbivore feedback that maintains savannas as alternative stable states [59,60], and large-scale regrowth has been shown to compensate for carbon losses from degradation across African woodlands [61]. Supporting the bidirectionality of these trends, McNicol et al. observed that large-scale regrowth often compensates for carbon losses from degradation in Africa [61]. Staver et al. found that African savannas are controlled by alternative stable states, with fire-induced feedback contributing to maintaining the coexistence of grass and trees [59]. This kind of fire–vegetation feedback can be strengthened by grazers, which can favor the increase in woody vegetation through a decrease in biomass fuel, hence lowering fire frequency, and therefore shifting the balance between grasses and woody plants [60].

In contrast with this bidirectionality, the net loss of SHR in EAF over the entire study period suggests unidirectional pressures for structural transformation favoring savanna grasslands at the expense of shrublands in this region. The trend that SHR losses in EAF are more channeled towards SHR→CRP than SHR→GRS at the transition level suggests that agricultural expansion rather than woody cover state change underlies SHR decline in EAF, in line with the rapid population growth and agricultural intensification of the Ethiopian highlands and Kenyan rangelands (corresponding to EAF in our work). Bullock et al. found that woody vegetation loss was overwhelmingly (35% increase) explained by transformation into CRP rather than fluctuation (regression into grassland/shrubland), a pattern that was confirmed for the continent by Ali et al. with small-scale agriculture [5,47].

We also observe the targeted transition of FST into SHR as well as the specific gain of CRP from SHR and GRS (SHR→CRP; GRS→CRP), consistent with a forest-to-agriculture cascade which can involve intermediate non-forest stages. This finding resonates with the degradation–deforestation continuum described by many studies, which emphasized that tropical forest loss does not take place abruptly but is frequently the result of a longer-term process going through several degradation stages, which has consequences on both the timing of carbon emissions and their reversibility [54,62,63].

### 4.5. Hydro-Climatic Dynamics: Wetlands and Surface Water in Africa

The shift in WET dynamics from historical gains to systematic decline, particularly via conversion to CRP in EAF, is consistent with reports of valley-bottom conversion for food production [64]. Globally, Davidson estimated that 64 to 71% of WET have been lost since 1900 [65]. More recently, Fluet-Chouinard et al. revealed that while WET loss has slowed worldwide, Africa emerges as an active hotspot of conversion, with the rate of loss increasing over the past 30 years and primarily attributed to the expansion of irrigated agriculture [66]. In the African context, however, much of this conversion is driven not by large-scale irrigation schemes but by smallholder recession agriculture and small-scale valley-bottom cultivation, where farmers exploit seasonally receding water levels for crop production [64].

The unidirectional WET→BAL transitions are consistent with persistent wetland degradation where disrupted hydrology triggers soil consolidation and loss of function [65,66]. The FST→WET transition warrants careful interpretation. Several mechanisms may explain it: (i) reservoir construction, as Africa is experiencing a hydropower boom with over 300 major dams planned or under construction, many inundating forested areas [67]; (ii) potential misclassification of newly established or transitional rice paddies as wetland, since flooded paddy fields are spectrally similar to wetland during the transplanting phase [68]; and (iii) genuine hydrological dynamics in gallery forest–riparian wetland mosaics of the Guinean zone [69]. Disentangling these mechanisms requires ancillary data (e.g., dam inventories, rice crop maps) beyond this continental-scale analysis.

The two-way WTR trend, with periods of active gain and loss in different regions, highlights the sensitivity of African surface waters to climate variability. The 2010–2015 loss phase, primarily in SAF and SAH, aligns with the initial phases of the intense 2015–2016 El Niño-induced drought when SAF experienced its driest summer rainfall season in 35 years [70]. This led to a significant reservoir drawdown and a large-scale desiccation of ephemeral water features.

### 4.6. Desertification Pathways and Recovery Constraints over Africa

The dormancy of BAL obscures several more targeted relationships that emerge from the transition-level analysis. The nearly constant GRS→BAL targeting and high contribution of SAH to this pathway indicate desertification processes that are active and even intensifying despite the broad pattern of Sahelian greening. This apparent contradiction of greenness at the regional scale with localized degradation is in agreement with Herrmann et al. and Kusserow, who found that although “regreening” is the principal signal at the regional scale, resulting from the recovery of rainfall patterns, this signal can mask “browning” hotspots of anthropogenic degradation [39,71]. Brandt et al. further warn that the greening signal may often result from the expansion of unpalatable woody species, rather than more functionally relevant ecosystem recovery, which could be why the GRS→BAL transition identifies a degradation signal not apparent in coarse-resolution vegetation indices [72].

The complete absence of BAL→GRS or BAL→WET targeting across the whole study period indicates severe constraints on recovery pathways for degraded lands. This asymmetric transition suggests that once vegetation cover is lost and bare soil is exposed, spontaneous recovery is almost impossible. This could only be possible through soil erosion, microclimate modification, or propagule limitation [73,74]. The significance of this pathway, and the degree to which it may be active in Africa, has major implications for land restoration: the United Nations Convention to Combat Desertification (UNCCD) Land Degradation Neutrality (LDN) framework posits that land degradation can be offset by land restoration at the same scale [75]. However, if BAL recovery is strongly constrained on decadal timescales, achieving Land Degradation Neutrality may require active intervention rather than assuming spontaneous recovery.

## 5. Conclusions

This study presents the first continental-scale long-term LC analysis of Africa by utilizing a spatially stratified Intensity Analysis framework to decode the spatiotemporal complexities of a rapidly transforming continent. Our results show a phase of LC change acceleration from 2010, mainly led by SAF, EAF, and WAF. At the category level, LC dynamics were defined by a distinct dichotomy between the volatility of GRS and SHR, expansion of CRP (intensity-wise) and loss of FST (area-wise). While SHR and GRS dominated gross turnover with SHR peak flux during 2010–2015, both maintained active status in gains and losses simultaneously. Critically, at this level, FST remained dormant throughout the study period despite being a consistent net loser. Smaller categories, such as WET and IMP, displayed the highest relative change intensities. WET dynamics shifted drastically from gains exceeding losses to net contraction concentrated in EAF. Our transition-level analysis uncovers that the apparent stability of African forests is deceptive. While net forest area changes can be qualified as dormant to moderate loss, a closer look at transition flows reveals a widespread regime of forest degradation (FST→SHR or FST→WET). Furthermore, the overall WET pattern shifted from historical net gains to recent systematic net losses, often involving irreversible transitions to BAL. Similarly, in the SAH, the persistence of targeted GRS to BAL transitions offers a necessary counter-narrative to the broad-scale greening hypothesis [39,72].

While this study provides a comprehensive quantification of LC dynamics, several limitations still need to be considered. This study characterizes the intensity, patterns, and magnitudes of LC change but does not quantitatively attribute these changes to their underlying drivers. Establishing causal linkages between LC transitions and their climatic or socioeconomic drivers would require spatially explicit regression or attribution modeling; therefore, deciphering the relative contributions from climatic change, demographic pressure, market integration and policy interventions remains beyond the scope of this study [26]. Moreover, the continental and sub-regional scale of this analysis may mask localized dynamics within individual ecological zones. For instance, the Guinean forests of West Africa have experienced well-documented degradation since the 1990s driven by oil palm expansion [32,51], yet these trends are diluted when aggregated within the broader WAF region alongside drier savanna zones. Prior regional studies have reported deforestation rates in specific Guinean forest blocks that substantially exceed the WAF regional average presented here [32]. The stratified framework adopted in this study represents a first-order disaggregation; finer ecological zonation would further improve the resolution of such localized trajectories. Future research should therefore integrate spatially explicit socioeconomic and climatic covariates to establish the connection between observed transitions and their drivers in Africa overall [33,76]. Furthermore, the reported accuracy of the GLC_FCS30D product (80.88%) means that some detected transitions may reflect classification error rather than genuine conversion. This is partially mitigated by (i) aggregating 35 classes into ten broad categories, which reduces confusion among spectrally similar sub-classes; (ii) using 5-year intervals as a temporal filter to suppress short-term classification noise [12]; and (iii) the Intensity Analysis framework itself, where ‘targeted’ transitions occurring at rates well above the uniform expectation are unlikely to arise from random misclassification. Nonetheless, transitions involving spectrally ambiguous classes (e.g., wetland boundaries, shrubland–grassland ecotones) should be interpreted with caution. Collectively, these findings underscore the need for spatially differentiated land management strategies across Africa, particularly the integration of Intensity Analysis-derived transition hotspots into national Land Degradation Neutrality targets, REDD+ monitoring frameworks, and regional agricultural development planning.

## Data Availability

To ensure full reproducibility, the complete computational framework is open-sourced at https://github.com/hidayathaider35/Africa-LC-Intensity-Analysis.git (accessed on 24 March 2026). This repository contains: (1) the Google Earth Engine scripts for generating transition matrices from the GLC_FCS30D product (projects/sat-io/open-datasets/GLC-FCS30D); (2) the Python implementation of the stratified Intensity Analysis framework; (3) all intermediate transition matrices used in this study; and (4) the supplementary results generation scripts for per-region analyses and temporal trajectory plots.

## Supplementary Materials

The following supporting information can be downloaded at: https://www.mdpi.com/article/10.3390/s26082318/s1, Figure S1: Interval-level intensity analysis for each IPCC AR5 sub-region: (a) EAF, (b) MED, (c) SAF, (d) SAH, (e) WAF. The left panel shows the gross change area per interval as a percentage of the regional map size. The right panel displays the annual change intensity compared to the regional uniform intensity (U); Figure S2: Category-level stratified intensity analysis for East Africa (EAF), 1985–2022. The left panel shows annual change area (km^2^/yr) and the right panel shows annual change intensity (%/yr). Solid bars represent gain; hatched bars represent loss. The red dashed line indicates the uniform intensity (U) for each interval (CRP: Cropland, FST: Forest, SHR: Shrubland, GRS: Grassland, TUD: Tundra, WET: Wetland, IMP: Impervious, BAL: Bare Area, WTR: Water Body, PSI: Snow/Ice); Figure S3: Category-level stratified intensity analysis for the Mediterranean region (MED), 1985–2022. See Figure S2 for legend description; Figure S4: Category-level stratified intensity analysis for Southern Africa (SAF), 1985–2022. See Figure S2 for legend description; Figure S5: Category-level stratified intensity analysis for the Sahara-Sahel region (SAH), 1985–2022. See Figure S2 for legend description; Figure S6: Category-level stratified intensity analysis for West Africa (WAF), 1985–2022. See Figure S2 for legend description; Figure S7: Transition-level stationarity matrices for each IPCC AR5 sub-region: (a) EAF, (b) MED, (c) SAF, (d) SAH, (e) WAF. Each off-diagonal cell contains eight strips representing time intervals from 1985–1990 to 2020–2022. Red strips indicate targeted transitions (R > W); grey strips indicate avoided transitions. Diagonal cells (hatched) represent persistence; Figure S8: LC class area change relative to 1985 baseline (%) for Africa and each IPCC AR5 sub-region. A value of 0% indicates no change from the 1985 area; positive values indicate expansion and negative values indicate contraction. Classes with less than 0.5% total change over the study period are excluded for clarity.
