# Supplementary material for: Significant Land Cover Transitions and Regional Acceleration at the Continental Scale of Africa over the Last Four Decades
Source: Sensors (Basel). 2026 Apr 9;26(8):2318. doi: 10.3390/s26082318 (PMC13119559; doi:10.3390/s26082318)
Supplement: Supplementary file 1 [file sensors-26-02318-s001.zip › sensors-4221555-supplementary.pdf]

Supplementary Material

for

Significant Land Cover Transitions and Regional Acceleration at the Continental Scale of Africa over the Last Four Decades

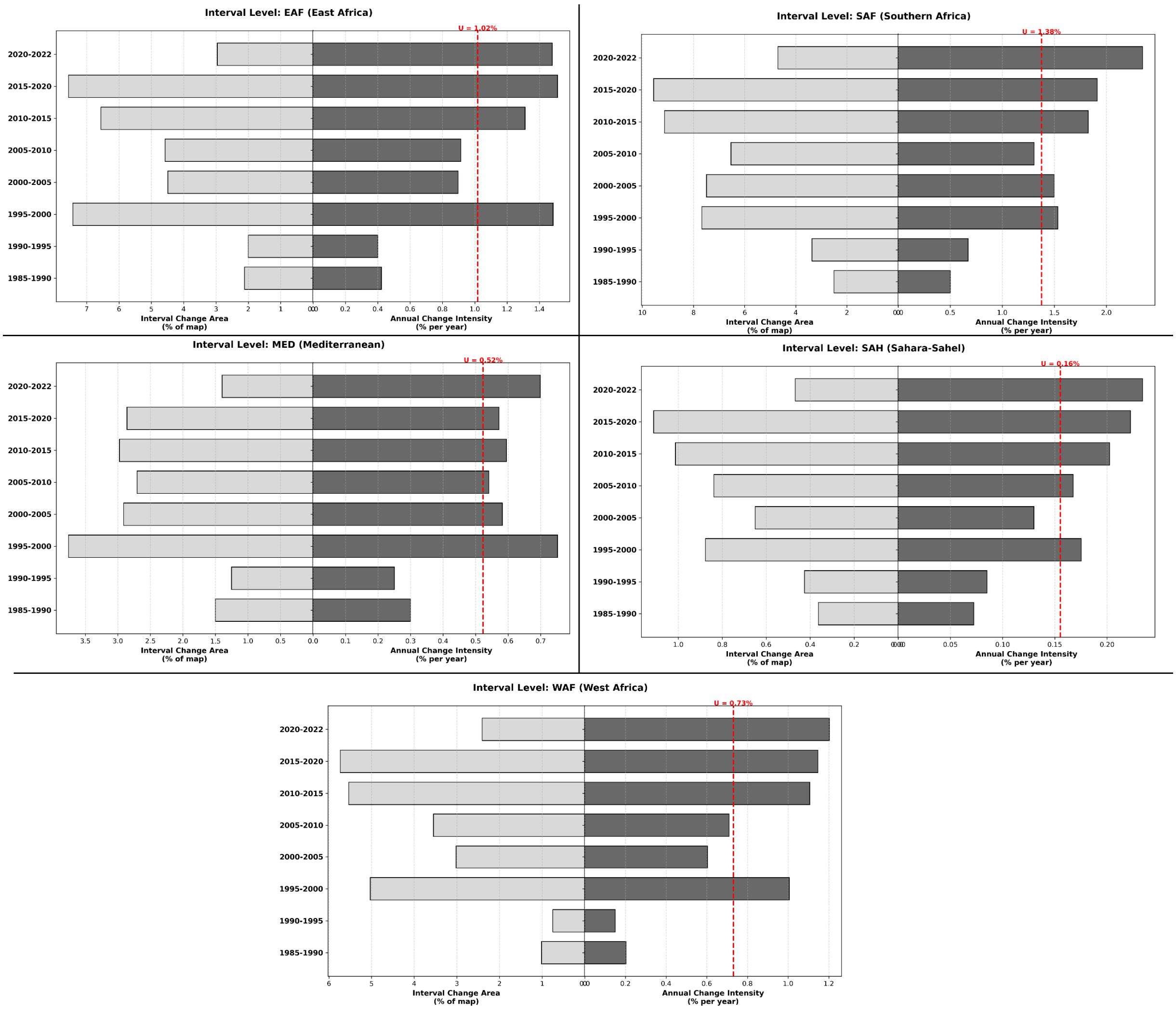

Figure S1. Interval-level intensity analysis for each IPCC AR5 sub-region: (a) EAF, (b) MED, (c) SAF, (d) SAH, (e) WAF. The left panel shows the gross change area per interval as a percentage of the regional map size. The right panel displays the annual change intensity compared to the regional uniform intensity (U).

EAF (East Africa) - Category Level

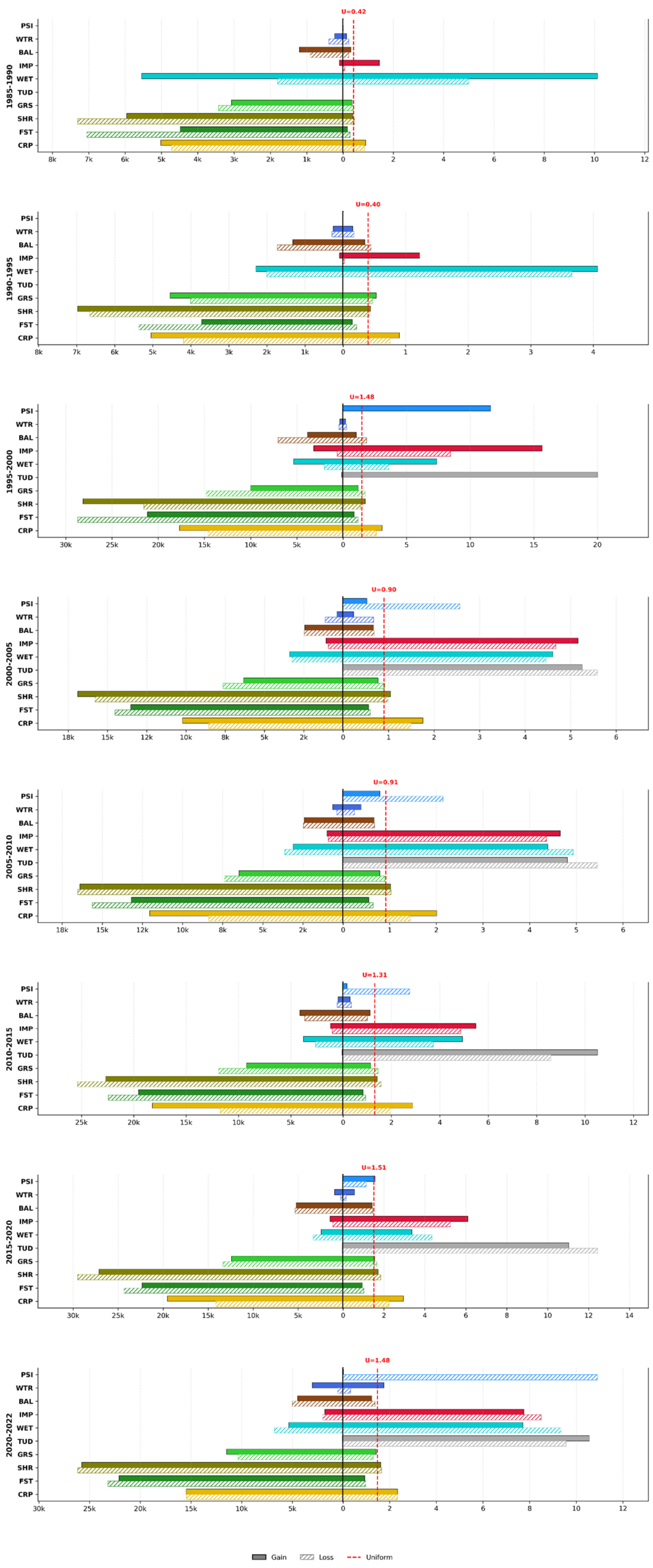

Figure S2. Category-level stratified intensity analysis for East Africa (EAF), 1985–2022. The left panel shows annual change area (km²/yr) and the right panel shows annual change intensity (%/yr). Solid bars represent gain; hatched bars represent loss. The red dashed line indicates the uniform intensity (U) for each interval (CRP: Cropland, FST: Forest, SHR: Shrubland, GRS: Grassland, TUD: Tundra, WET: Wetland, IMP: Impervious, BAL: Bare Area, WTR: Water Body, PSI: Snow/Ice).

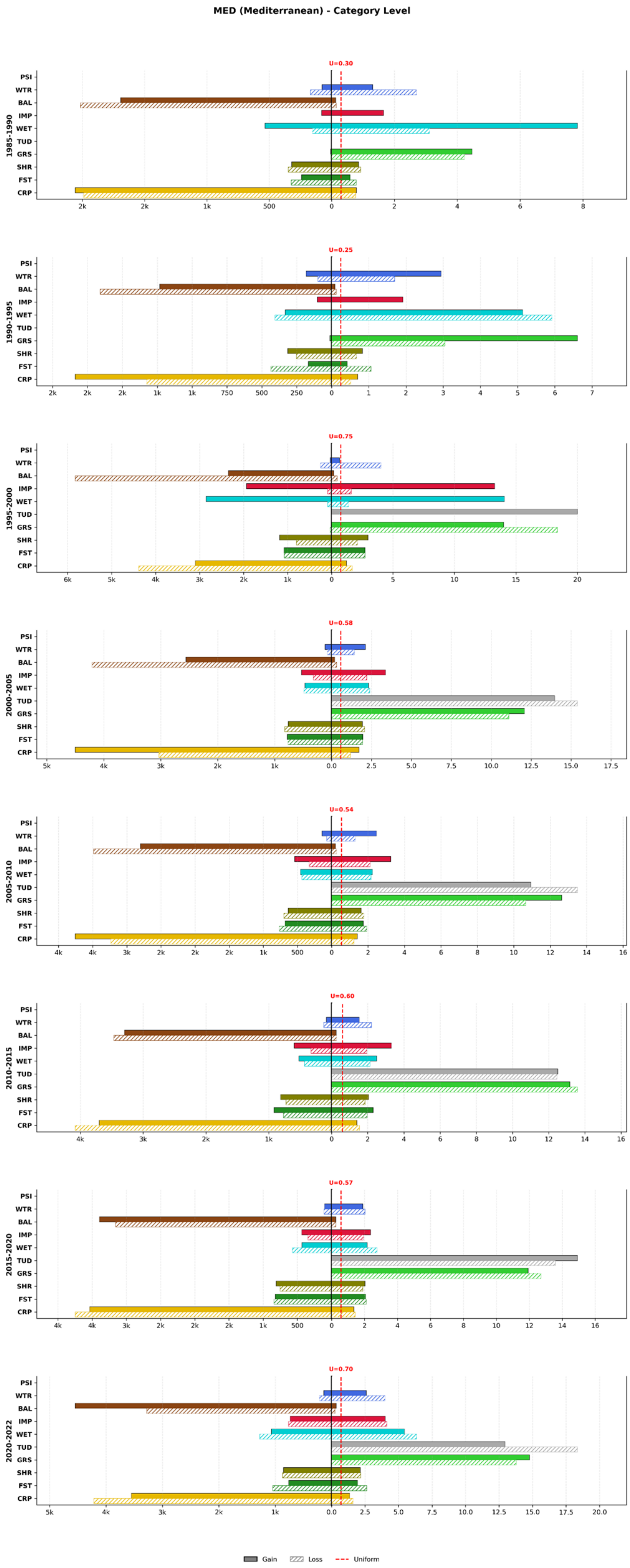

Figure S3. Category-level stratified intensity analysis for the Mediterranean region (MED), 1985–2022. See Figure S2 for legend description.

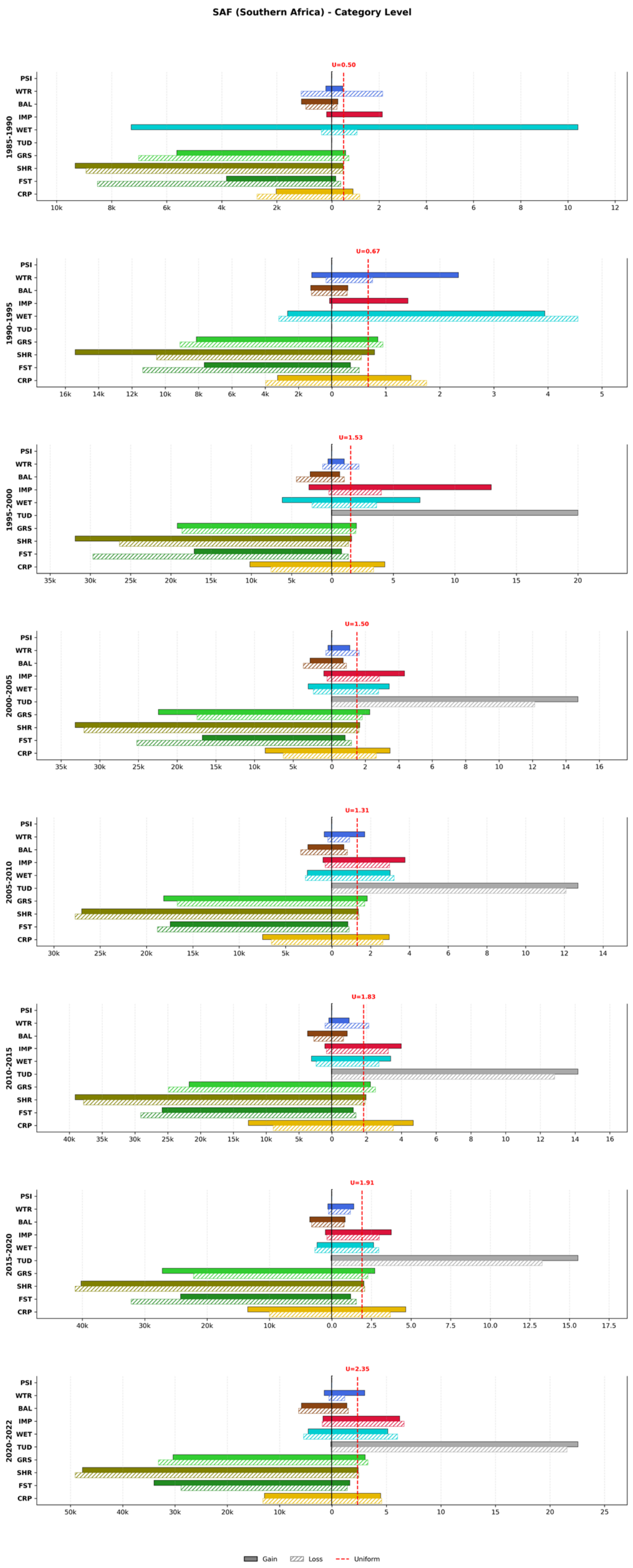

Figure S4. Category-level stratified intensity analysis for Southern Africa (SAF), 1985–2022. See Figure S2 for legend description.

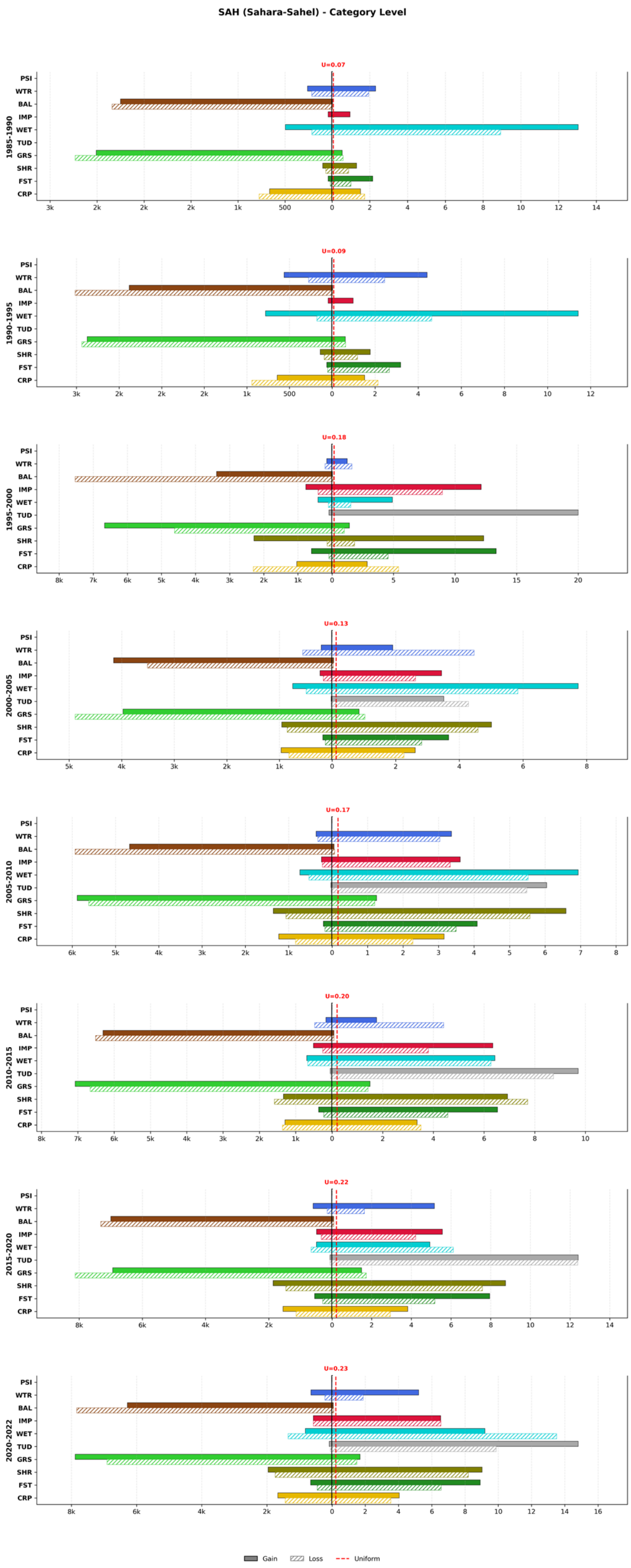

Figure S5. Category-level stratified intensity analysis for the Sahara-Sahel region (SAH), 1985–2022. See Figure S2 for legend description.

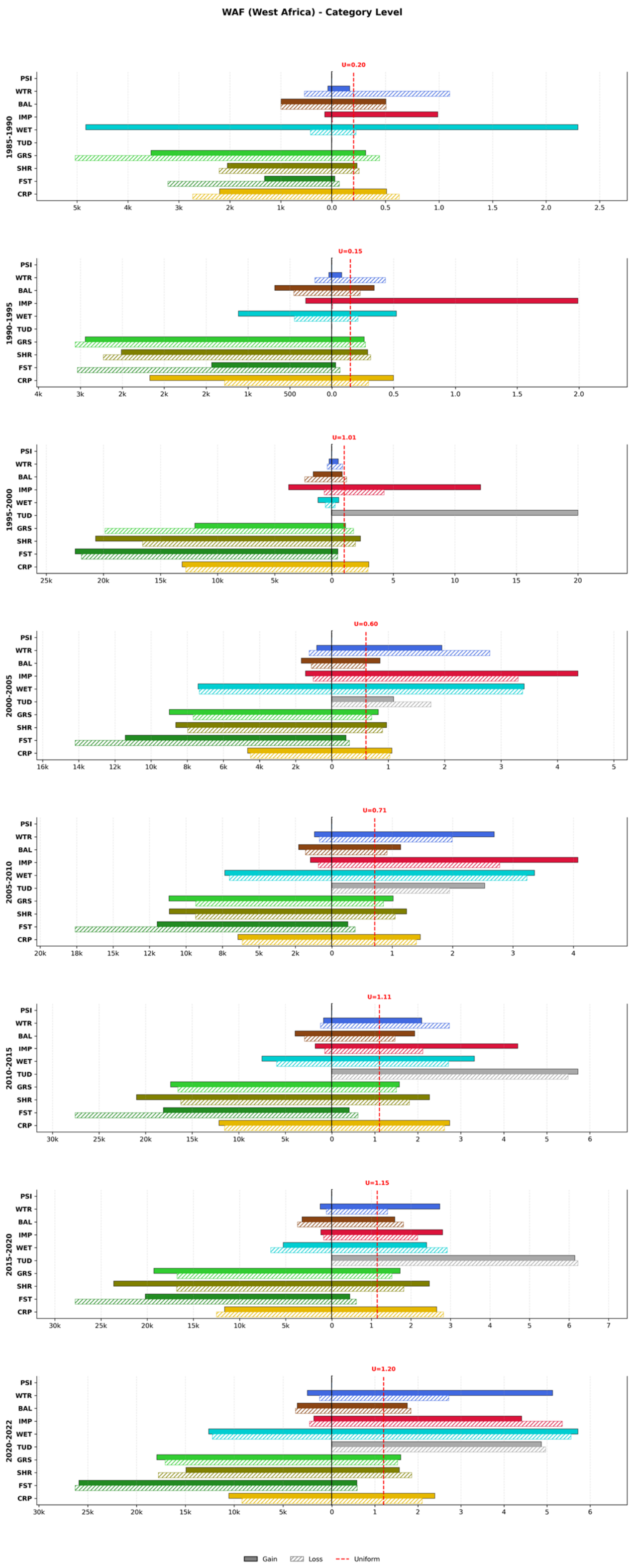

Figure S6. Category-level stratified intensity analysis for West Africa (WAF), 1985–2022. See Figure S2 for legend description.

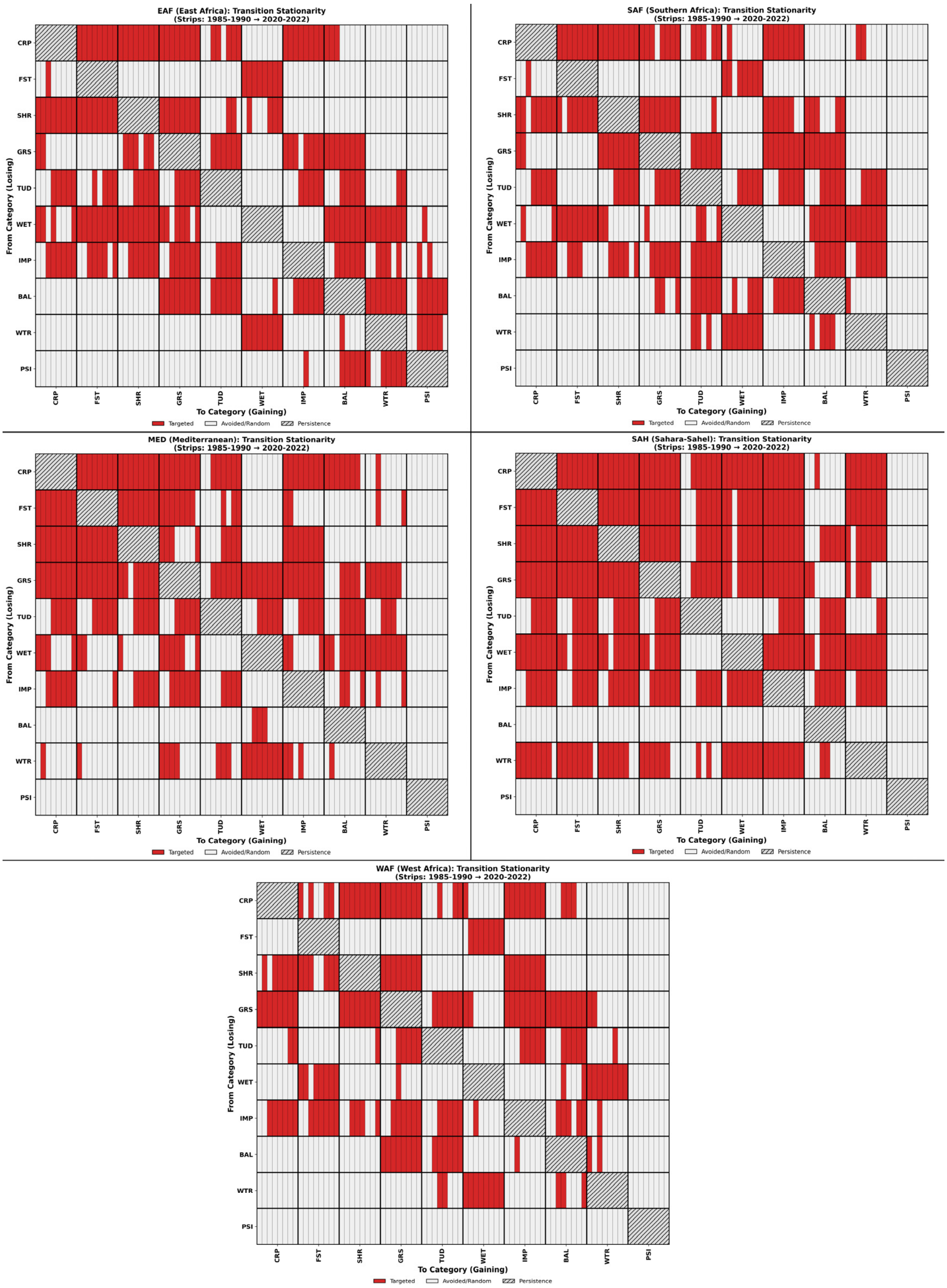

Figure S7. Transition-level stationarity matrices for each IPCC AR5 sub-region: (a) EAF, (b) MED, (c) SAF, (d) SAH, (e) WAF. Each off-diagonal cell contains eight strips representing time intervals from 1985–1990 to 2020–2022. Red strips indicate targeted transitions (R > W); grey strips indicate avoided transitions. Diagonal cells (hatched) represent persistence.

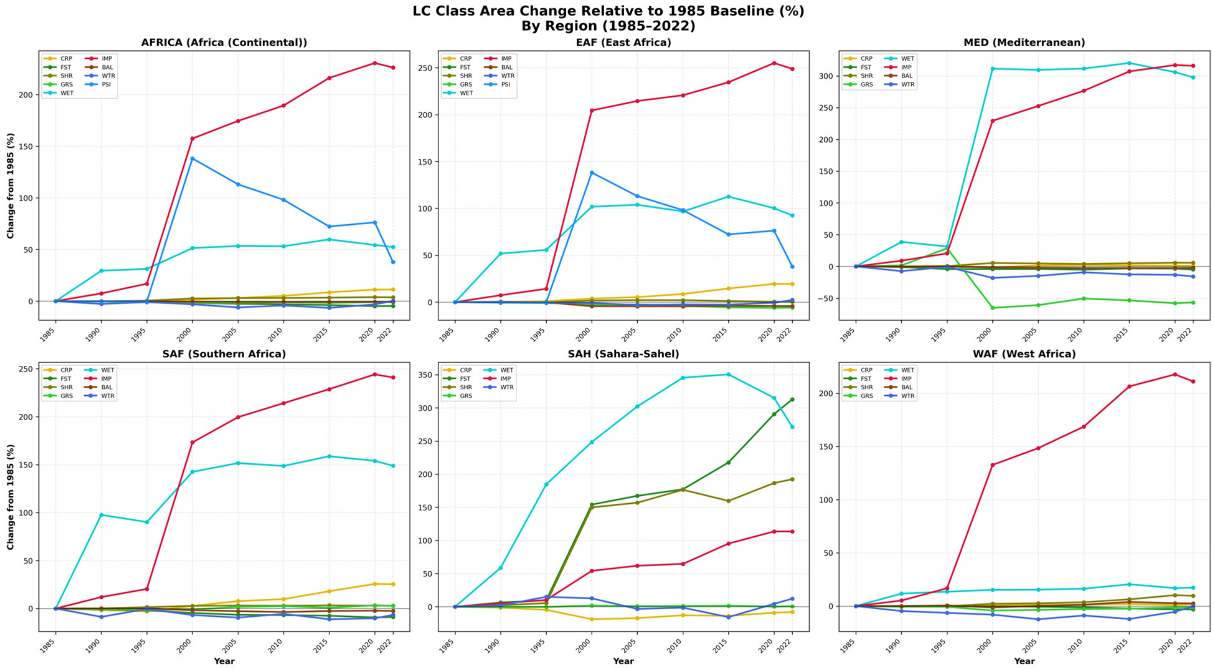

Figure S8. LC class area change relative to 1985 baseline (%) for Africa and each IPCC AR5 sub-region. A value of 0% indicates no change from the 1985 area; positive values indicate expansion and negative values indicate contraction. Classes with less than 0.5% total change over the study period are excluded for clarity.
